# Clinical Consultation as a Family Intervention in Residential Treatment: Exploring What Impacts Outcomes

**DOI:** 10.1007/s10591-015-9365-3

**Published:** 2015-12-08

**Authors:** Catherine McConnell, Patricia Taglione

**Affiliations:** Allendale Association, PO Box 1088, Lake Villa, IL 60046 USA

**Keywords:** Residential treatment, Family involvement, Clinical consultation, Therapeutic alliance

## Abstract

The Relational Re-enactment Systems Approach to Treatment model is an approach to residential treatment that embraces the need for family involvement through clinical consultation. Clinical consultation is a systems-oriented family intervention that embodies the model’s principles regarding therapeutic alliance and working through ambivalence. Families engage with treatment providers and other collaterals in an ongoing process of developing goals, creating a shared understanding of the youth, and working toward discharge. The current study explored youth characteristics and outcomes. Additionally, the investigation included comparisons between youth with and without the involvement of the Department of Children and Family Services in terms of length of stay, involvement in consultation, and sustained outcomes. Finally, therapists who work with youth and their families discussed their understanding of what differentiated successful and unsuccessful cases.

## Introduction

There is little debate that family involvement for youth in residential treatment is not only a research-based facet of effective treatment but also a humane component of working with youth and their families (Geurts et al. 2012; Hair 2005; Walter and Petr 2008). The utility of residential treatment as a part of the continuum of care for youth is sometimes challenged (Barth 2005; Frensch and Cameron 2002), but for those youth who are placed residentially, their life after discharge seems impacted by the role their family played in their treatment.

Although the importance of working with families is accepted, the nature of this involvement is still being investigated. Family therapy is only one method of involving families in their child’s treatment, and has evolved in some cases to better meet the needs of families (Huefner et al. 2015). For example, in order to make services accessible to families, therapy by phone has been used (Robst et al. 2013). The options appear broad for facilitating a family’s role in its youth’s care. Given this breadth of possibility, there is question about what makes family involvement optimally successful. While there is some evidence that almost any involvement—even if it is not specifically therapeutic—is beneficial, the need to provide therapeutic interventions to families and other caregivers who will remain in the lives of youth with mental health challenges seems crucial (Lakin et al. 2004).

The Relational Re-Enactment Systems Approach to Treatment (REStArT) is a model for residential treatment that seeks specifically to address the challenges inherent in engaging families in their youths’ care. While the introduction of this intervention at one agency precipitated improvements in outcomes broadly (McConnell and Taglione 2012), the current study was intended to explore the aspects of treatment in aggregate, either case characteristics inherent to particular subsets of youths and their families or program dynamics, that differentiated youth who were successful in the program. This exploration included an examination of both the frequency of family participation as well as the process of the family involvement. Additionally, previous research had looked at outcomes at the time youth were discharged, whereas the current study considered youth’s ability to maintain his or her placement in the months following discharge as well. By studying the characteristics of successful cases, we hoped to better understand the factors that encourage or possibly impede family involvement as a catalyst for positive youth outcomes.

## The REStArT Model and Clinical Consultation

The REStArT model originated at a multi-service agency for youth and their families that provides residential treatment. Within the agency’s residential program, the “high-end” treatment units are unlocked but considered the most restrictive level of care for youth whose behavior can no longer safely be addressed in the community. Since the model’s implementation in 2007, youth at one agency increasingly saw positive outcomes to less restrictive levels of care (McConnell and Taglione 2012). The model provides a coherent yet flexible approach to treatment that is informed by research on trauma-based treatment, attachment and developmental theories, systems theory and object-relations (Taglione et al. 2014). Thirteen guiding principles articulate the tenets of the model and provide the basis for training (see “Appendix”). Additionally, the principles support the model’s primary intervention for working with families: clinical consultation.

### Principles of the Model

Presented here is a very brief overview of some of the model’s principles that are most relevant to working with families. The REStArT training manual (Taglione et al. 2014) includes a broader description of the model’s principles, core concepts, and interventions than the scope of this paper allows (the manual is available from the authors on request). The first principle of the model and the one most central to consultations is the treatment providers’ therapeutic alliance with youth and their families. The therapeutic alliance is often defined not simply as the quality of the relationship between clinician and client but as agreement on the goals of treatment as well as the process of obtaining those goals (e.g., Norcross and Wampold 2011). This alliance appears to be a part of evidence-based therapy relationships, both individual and family (Friedlander et al. 2011; Shirk et al. 2011).

Developing a balanced therapeutic alliance in family treatment is difficult even outside the residential treatment setting due to the multiple relationships in the treatment process (Hogue et al. 2006; Robbins et al. 2003). But the therapeutic alliance is further complicated in residential treatment by not only multiple family members participating in treatment, but also multiple providers, sometimes from multiple agencies. The training and ongoing supervision involved in the model’s implementation contain decision trees that guide providers through the process of assessing the alliance with youth and their families.

Sometimes, even when clients have articulated goals for themselves and appear committed to those goals—for example, to return to their family of origin or to move on to independence—they may still have ambivalence. In residential treatment, much like family work in other therapeutic settings, ambivalence is sometimes expressed through clients acting in ways that elicit a response in treatment providers to “take the other side” of the ambivalence (Miller and Rollnick 2013). The emphasis in the model on ongoing feedback provided by supervisors and colleagues allows therapists and other treatment providers to be cognizant of the pull to persuade family members. As a result of this reflection, providers can be aware of what factors may be motivating both sides of the family’s ambivalence and give them the space in the family work to resolve the ambivalence in ways that are authentic to them. In doing this, therapists and others also activate the principle of the model that asks providers to expect health from youth and their families.

Being aware of how youth or their families may be engaging us in the process of avoiding their ambivalence is only one of the ways that self-reflection is used in the REStArT model. The model extends the work of Wood and Long (1991) in using “conflict cycles” to better understand the re-enactments that occur between youth and adults. The conflict cycle provides a unique understanding of each youth by identifying their individual stressors and as well as their individual ways of emotionally and behaviorally responding to those stressors. The conflict cycle also includes the adults’ ways of responding to youth behaviors. Often these adult responses inadvertently maintain the youth’s conflict cycle either by amplifying the original stressor (a counter-aggressive response) or by protecting the youth from the stressor (a counter-indulgent response). Both counter-responses interfere with the youth’s opportunity to develop new and more adaptive ways of responding to their own stressors.

The understanding of an individual youth’s conflict cycle requires treatment providers to be aware of their counter-response, which may be indulgent (that is, feeling overly empathic and excessively externalizing the youth’s presenting problem) or aggressive (such as feeling frustrated with lack of progress). Due to the histories of trauma that most of the youth in residential treatment have and the conduct disordered behavior that has then precipitated their placement, many of the children and adolescents in treatment present with a “control-sensitive” conflict cycle; that is, one in which they may alternately view themselves as victims or aggressors. They may elicit complementary feelings in the adults working with them that, in turn, can result in overly permissive or excessively controlling approaches to working with the youth. The understanding of re-enactments as conflict cycles, managing counter-response, and developing plans to interrupt the cycle are the focus of three of the model’s principles.

### Clinical Consultation

Clinical consultation, one of the main interventions of the REStArT model, was originally developed to address deficits in engaging families in their youths’ care. Clinical consultation involves regularly scheduled collaborative contact between members of the youth’s treatment team, the youth, and his or her family. Caseworkers from funding sources as well as other collaterals are also encouraged to participate in the process. Especially for youth who have funding through a state child welfare agency, the external caseworker for that youth is considered an integral part of the consultation process because of the youth’s legal standing with the state. Consultations are scheduled at the convenience of the family and more often than not take place via telephone in order to accommodate work schedules and travel distance. Unlike a more traditional approach to consultation, clinical consultation does not position the treatment providers as the authorities and the family as recipients of the providers’ expertise and direction. Nor are these contacts an opportunity for staff simply to inform family members or collaterals about the youth’s progress. Rather, consultations are intended to address multiple principles of the model.

On center stage during the consultation process is the assessment of the therapeutic alliance between youth, family, and treatment providers. As mentioned above, the alliance between family and providers is even more complex in residential treatment than it may be in a traditional family therapy setting. Families encounter many providers within milieu treatment. It is this complex system that clinical consultation seeks to balance, in part by addressing in each clinical consultation the goals for discharge that the youth and his or her family have identified even if those goals differ from what the providers think they should be. Additionally, clinical consultation removes the compartmentalization of relationships between family members and different providers within the agency that may make it more likely for splits to develop. Therapists and other providers are asked to look for evidence of the strength of the alliance in each consultation meeting.

Clinical consultation also provides a forum to address ambivalence about treatment and discharge goals that youth or families may be experiencing. Clinical consultation requires providers who are facilitating the consultation to be aware of this ambivalence and watch for ways that it may be acted out rather than explicitly communicated. Because progress toward discharge is a regular focus of clinical consultation, providers have the opportunity to consistently assess and bring to the attention of youth and families discrepancies between their stated goals and their progress toward those goals.

The importance of the youths’ conflict cycles in clinical consultation cannot be overstated. The conflict cycle provides the opportunity for therapists, caseworkers and family members to create a shared understanding of the youth. This shared understanding also addresses a criticism sometimes leveled at residential treatment providers by families that family members feel blamed by their youths’ providers (c.f., Walter and Petr 2008). Additionally, the conflict cycle offers a rationale for the ongoing structure that youth may need when they return to a less restrictive environment, which helps families see the importance of this.

## The REStArT Model: A Case Example

A case that illustrates the implementation of REStArT model and its family intervention is that of Carlos, a 15-year-old Latino adolescent who came to residential treatment following many years of out-of-home placements such as psychiatric hospitalizations, state mental health hospitals, and group homes. He had a history of aggression against other children and adults, including a security guard at a hospital. He also engaged in self-harm that included ingesting foreign objects. Additionally, he made what were deemed to be false accusations against others. Carlos’ medical history was complex, leading him at times to claim that he needed hospitalization even when it was not necessary. Furthermore, Carlos’ brother had died when Carlos was young and he appeared to be re-enacting the trauma of this loss as well as fears about his own mortality through his repeated medical hospitalizations. Clinical consultations included the treatment team as well as Carlos, his mother, and the caseworker from his funding source. At the time that he was admitted, Carlos’ mother was identified as his discharge resource but his funding source did not agree with this goal.

Despite wanting to be discharged from residential treatment, Carlos often made allegations against others and requested hospitalizations. During consultation, the treatment team started to point out the ambivalence that was emerging: on the one hand, he wanted to return home to his mother and have more freedom, but another part of him may have sought the structure that was provided by the hospital and acted out in ways that ensured he would stay in residential care. Carlos’ mother also appeared to be ambivalent about having him return home. The treatment team identified that Carlos and his mother sometimes avoided addressing their ambivalence about one another by joining together in conflict with treatment providers. Interventions to address this ambivalence started with the team’s awareness of ways in which they felt pulled to either indulge requests made by Carlos and his mother in order to avoid conflict or to withhold reasonable requests out of frustration with their many demands. By interrupting this cycle of conflict, Carlos and his mother were then able to engage with the treatment team in their alliance of working together to have Carlos safely discharged. This process also helped Carlos and his mother see ways that he would need to continue to have structure in place even if he was no longer in treatment.

As his acting out subsided, however, his funding source still did not support his placement with his mother but rather wanted him to step down to a group home. Although staff were aware of their wish to take on this battle for Carlos and his mother and maybe try to convince the funding source that Carlos should return home, the team instead sought a more balanced response that allowed Carlos and his mother to work with the funding source more realistically. Carlos, wanting to leave even if he didn’t go home, eventually agreed to go to the group home. This step down was, therefore, technically a positive outcome but was not sustained. Soon after being placed in the group home, Carlos ran away from treatment. But rather than running to the hospital as he had in the past, he ran to his mother. He remains stable and with his mother over 2 years later.

## Methods

Research in residential treatment is inherently complex (Curry 2004). As such, our approach to our questions about family interventions and outcomes was exploratory in that we assumed family involvement would be an important factor in successful treatment but did not have specific hypotheses about the nature of this relationship. We began with aggregate descriptive data to look at group differences in outcomes. We then sought qualitative descriptions of successful and unsuccessful cases from therapists in order to create a more three-dimensional look at youth and family’s experiences in the program. The processes for both the quantitative and qualitative components of the study follow.

### Youth Sample

The agency involved in this study is a comprehensive treatment facility for youth and their families. The “high-end” residential program—that is, the most restrictive of the residential programs—has a capacity of approximately 130 youth at any given time. We selected as our sample all youth who were discharged in the fiscal year 2014 from the high-end treatment program. The youth in this program either have a conduct disorder diagnosis or have exhibited behaviors that are related to conduct disorder that cannot be contained in a less restrictive environment. These behaviors are the precipitants to placement, although additional diagnoses may include mood disorders, posttraumatic stress disorder, autism spectrum disorders, and borderline personality disorder. The selection of this particular group allowed for a sample that was recent enough to reasonably reflect the current implementation of the model but also allowed for a sample from which we would have information on whether discharges that were favorable had been sustained for 6 months.

In this current sample, there were 100 discharged youth who ranged in age from 13-years-old to 21-years-old, with an average age at discharge of approximately 17 years (*M* = 16.76, *SD* = 1.51). Most of the youth discharged were boys (68 %) and the majority of the youth were African American (56 %). An additional 34 % of the youth identified as white, 7 % as Latino/a and 3 % as bi-racial. The Department of Child and Family Services (DCFS) was the funding source for and, therefore, also the legal guardian of 66 % of the youth. The remaining 34 % had an alternative funding source and a guardian who was either a biological family member or adopted family. These clients had state School Board of Education funding (18 %), Department of Human Services funding (13 %) or funding through a neighboring state (3 %). Table 1 provides a description of the sample.

**Table 1:** Sample characteristics

	%	*M*	*SD*
Gender			
Male	68		
Female	32		
Age (in years)		16.76	1.51
Race			
African American	56		
White	34		
Latino/a	7		
Bi-racial	3		
Funding source			
DCFS	66		
DHS	13		
ISBE	18		
Wisconsin	3		
Type of discharge			
Favorable/sustained	68		
Favorable/not sustained	14		
Unfavorable	18		
Length of stay (in months)		13.18	6.92
Clinical consultation rate (consults per month)		1.20	0.75

Characteristics of all youth (*N* = 100) and their discharges during the 2014 fiscal year. DCFS is the Department of Children and Family Services. DHS is the Department of Human Services. ISBE is the state board of education. Wisconsin refers to funding through that state’s mental health services. Favorable/sustained discharges are those that are positive (family home, group home, transitional living programs, moderate residential programs, independence, foster care, and less restrictive adult mental health agencies) and also sustained at 6 months. Favorable/not sustained are discharges that were positive at the time of discharge but had disrupted by 6 months post-discharge. Unfavorable discharges were negative outcomes (runaway, detention, psychiatric hospital or transfer to another residential program)

### Therapist Participants

Six therapists were asked to participate in the qualitative portion of the study and all six agreed to participate. All were therapists at the agency during the fiscal year 2014 in which the youth for this study were discharged. They had all received ongoing training on the implementation of the REStArT model. Four of the six therapists were female. They had an average of 4.8 years of experience, with a range of 3–8 years as therapists at the agency. Each therapist worked primarily with a single residential unit and they provided individual therapy to the youth in this unit. Therapists also facilitated the clinical consultation intervention with the cooperation of the youth’s agency caseworker.

### Variables

Discharge outcome as a dependent variable was assessed both at the time youth exited the program and also after 6 months. A “favorable” discharge was one in which the youth left the high-end residential program for a less restrictive level of care including a group home (this program’s or another’s), family home, foster care, a transitional living program, a more moderate residential program, independence or a less-restrictive adult mental health facility. Lateral discharges to other residential programs or psychiatric hospitals as well as more obviously negative outcomes such as detention and runaways who did not return to the program were considered “unfavorable” discharges.

The agency routinely offers after-care consultation to youth and their families or caretakers following program discharge, and this also allows us to track whether youth have maintained their placement. For this study, we measured sustained outcomes at 6 months. Because we consider the youth’s ability to maintain his or her placement as a measure of our alliance with the youth and his or her family, for the purposes of this study, we delineated our sample as follows: “positive” outcomes were those that were favorable and sustained at 6 months and “negative” outcomes were those that were either unfavorable at the time of discharge or were not sustained for 6 months. This was conceptually congruent for our model but it also allowed for “oversampling” of unfavorable discharges as our favorable results proportion at the time of discharge is high and would create uneven sub-samples.

Independent variables that we examined for their relationship to discharge status included rate of consultations (average number of consultations occurring in each month of stay), length of stay, and funding source. The rate of consultations was selected in order to determine whether frequency of this particular form of family contact influenced outcomes, especially in light of previous research suggesting that family involvement and family therapy are most impactful on residential outcomes (Robst et al. 2013). Although it is not clear from previous research if length of stay is associated with deleterious effects for the youth in treatment, it was selected as a variable due to increased focus on its impact on treatment (James et al. 2012). Length of stay was measured by the number of months the youth was placed in the agency’s high-end program. Funding source was divided between youth who were funded by the Department of Children and Family Services and youth funded by all other sources which included the Department of Human Services and the state School Board of Education. This variable stands in for the different case dynamics present in working with youth whose families are their legal guardians compared to youth for whom the state is their guardian.

### Data Analysis

#### Quantitative Analysis

Consultation rate, length of stay, and funding source were all assessed for their relationship to outcomes 6 months post-discharge. Separate two-tailed *t* tests assuming unequal variance were conducted to measure the difference between the mean consultation rate by outcome type and between the mean length of stay by outcome type. As an exploratory study, we were interested in their independent relationship with outcomes rather than their combined explanation of variance. A Chi square test was used to assess the difference between the proportion of positive and negative discharges by funding source. Because this Chi square test produced the only significant relationship between one of the variables (funding source) and outcomes, we also conducted t-tests for the mean consultation rate and mean length of stay by funding source.

#### Qualitative Analysis

Because, with few exceptions, the negative outcomes in this sample were youth who were funded by DCFS, we also conducted a qualitative review of the model’s implementation across selected cases. Residential therapists of the units whose youth were included in the discharge sample were asked to provide recollections about the process of consultation with DCFS youth and their families. In particular, we were interested in exploring the ways in which the model’s primary family intervention, clinical consultation, impacted differential outcomes. Therapists were provided with a list of DCFS youth who had discharged in the fiscal year 2014. Therapists selected two cases to discuss, one with a positive and one with a negative outcome, based on their ability to recall details about the process of the youth’s treatment in those cases. They were then asked to respond in writing to the question, “What aspects of treatment, especially related to consultation, did you notice seemed to help or hinder the case?” The therapists were instructed to focus on the process of clinical consultation because we were specifically interested in their reflections on family involvement. The written responses from therapists were separated into groups by discharge outcome. Narratives were assessed for themes related to REStArT model principles and were considered salient if they occurred across a majority of respondents.

## Results and Discussion

### Descriptive

Most youth discharged in fiscal year 2014 had a favorable outcome at the time of discharge (82 %, *n* = 82). Although it is difficult to compare outcome studies to one another given different populations and methodologies, this rate of favorable discharges appears comparable to other favorable studies (c.f., Hair 2005; Thomson et al. 2011). Most favorable discharge outcomes for the youth in this sample were either to a family home or to a group home (25 and 31 % of total discharges, respectively). The remaining favorable discharges were to transitional living programs (10 %), moderate residential treatment (7 %), foster care (6 %), adult mental health programs (2 %) or independence (1 %).

Youth with DCFS funding accounted for 48 % of the discharges to family homes and this represented 18 % of all DCFS-funded discharges. Alternately, 34 % of youth without DCFS funding returned home. The unfavorable outcomes at the time of discharge included runaway (11 % of total discharges), detention (5 %) and lateral moves to other high-end residential programs (2 %). All of the discharges that were due to runaways and detention were youth who had DCFS funding. Table 2 contains discharge type by funding source.

**Table 2:** Discharge distribution by funding source

	Total (*N* = 100)	DCFS (*n* = 66)		Non-DCFS (*n* = 34)	
		At discharge	Sustained at 6 months	At discharge	Sustained at 6 months
Favorable discharges	**82**	**49**	**38**	**33**	**30**
Group home	31	14	12	17	16
Family home	25	12	9	13	12
TLP	10	9	5	1	0
Moderate residential	7	7	6	0	0
Foster care	6	6	5	0	0
Adult mental health	2	0	0	2	2
Independence	1	1	1	0	0
Unfavorable discharges	**18**	**17**		**1**	
Runaway	11	11		0	
Detention	5	5		0	
Lateral residential	2	1		1	

Discharge status for youth overall (*N* = 100) and by funding source at the time of discharge and sustained at 6 months. DCFS is Department of Children and Family Services. Non-DCFS is all other funding sources including Department of Human Services, the state school board of education, the state department of corrections, and a neighboring state’s funding for mental health services

The bold values indicate subheading totals

Of the 82 favorable discharges, 83 % (*n* = 68) were sustained at 6-months. Therefore, from the original sample of 100 discharges, 68 % were positive at 6-months. The remaining 32 % were negative, meaning they were either unfavorable at discharge (*n* = 18) or were not sustained or unknown at 6-months (*n* = 14). Of the discharges that were not sustained, four of 25 discharges to family homes disrupted, three of 31 group home discharges disrupted, and half of discharges to transitional living program disrupted (five of 10). Three of the favorable discharges were considered negative outcomes at 6 months because their disposition was unknown. These included one foster care discharge and two discharges to family homes.

### Quantitative

As the primary family intervention, clinical consultation is offered to all youth and families. In the current sample, 95 % of the youth and their families participated in clinical consultation during their treatment. Youth who had positive outcomes had, on average, fewer consultations per month (*M* = 1.17, *SD* = 0.69) than youth with negative outcomes (*M* = 1.26, *SD* = 0.87). This difference in rate of consultations was not statistically significant, nor was it practically meaningful (see Table 3 for differences between outcomes). Both youth with positive outcomes and those with negative outcomes were participating in approximately one to two consults for each month they were in treatment. Although there was a statistically significant difference between rates of consultation for DCFS youth and non-DCFS youth (*t* = −3.63, *p* < 0.0006), the actual difference was an average of half a consultation a month (*M* = 1.00, *SD* = 0.64 and *M* = 1.58, *SD* = 0.81, respectively). While this may represent a larger issue about how DCFS youth were approached, the actual difference is small (see Table 4 for differences between funding sources).

**Table 3:** Differences between positive and negative outcomes

	Positive outcome at 6 months			Negative outcome at 6 months			*t*	*p*
	*n*	*M*	*SD*	*n*	*M*	*SD*		
Length of stay (in months)	68	13.47	7.41	32	12.56	5.78	0.67	0.51
Rate of consultations (consults per month)	68	1.17	0.69	32	1.26	0.87	−0.48	0.63

Differences between positive and negative outcomes by mean length of stay and mean rate of consultations. Positive outcomes at 6 months were discharges that were positive (family home, group home, transitional living programs, moderate residential programs, independence, foster care, and less restrictive adult mental health agencies) and also sustained at 6 months. Negative outcomes at 6 months were either positive at the time of discharge but not sustained or were negative at the time of discharges (runaway, detention, psychiatric hospital or transfer to another residential program)

**Table 4:** Differences between funding sources

	DCFS			Non-DCFS			*t*	*p*
	*n*	*M*	*SD*	*n*	*M*	*SD*		
Length of stay (in months)	66	14.14	6.23	34	11.32	7.85	1.82	0.07
Rate of consultations (consults per month)	66	1.00	0.64	34	1.58	0.81	−3.63	0.000

Differences between positive and negative outcomes by mean length of stay and mean rate of consultations. DCFS is Department of Children and Family Services. Non-DCFS is all other funding sources including Department of Human Services, the state school board of education, the state department of corrections, and a neighboring state’s funding for mental health services

It was somewhat surprising to find that there was no relevant difference in the amount of time spent with families related to discharge outcomes considering previous research that has found such a link (Huefner et al. 2015). This suggests that the agency was equally involving youth and their families in treatment regardless of outcomes, implying that variables other than simply the quantity of treatment were at play in the relationship between family contact and the maintenance of favorable discharges. Notably, of the five youth who did not participate in any family intervention during their stay, three youth were discharged due to running away from the program.

The average length of time in treatment for all clients was 13.18 months (*SD* = 6.92), with a minimum stay of 2 months and a maximum stay of over 3 years (39 months). Length of stay did not differ between positive and negative outcomes (*M* = 13.47 months, *SD* = 7.41 and *M* = 12.56 months, *SD* = 5.78 respectively). The length of stay for youth with DCFS funding was 14.14 months (*SD* = 6.23) and the length of stay for youth with an alternative funding source was 11.32 months (*SD* = 7.85). The difference between these two groups was not statistically significant (*t* = 1.81, *p* < 0.07); however, the difference may still be meaningful in that DCFS youth were spending almost 3 months more in treatment. Longer lengths of stay have not always been associated with an actual need for a longer stay but rather the absence of a post-discharge placement option (James et al. 2012).

The proportion of positive and negative discharges varied significantly by funding source, *X*^2^ (1, *N* = 100) = 9.69, *p* = .0002 (see Table 5). Youth with DCFS funding represented two-thirds of the youth discharged but were just over half of the sustained positive discharges. On the other hand, only four of the 34 youth without DCFS funding had negative discharges (meaning, discharges that were either unfavorable or not sustained at 6 months). Certainly some of this difference is apparent in how many returned to a family home. Of the 68 DCFS-related discharges, 12 returned to a family home (17.6 %), and nine of these youth were still in that home at 6 months. On the other hand, 13 of the 34 youth without DCFS funding (38.2 %) returned home and only one was not sustained at 6 months.

**Table 5:** Proportion of positive discharges by funding source

	*n*	Positive outcome at 6 months (%)	*n*	Negative outcome at 6 months (%)	Total
DCFS funding	38	57.6	28	42.4	66
Non-DCFS funding	28	87.5	4	12.5	32

*X* ^2^ (1, *N* = 100) = 9.69, *p* = .002

Positive outcomes at 6 months were discharges that were positive (family home, group home, transitional living programs, moderate residential programs, independence, foster care, and less restrictive adult mental health agencies) and also sustained at 6 months. Negative outcomes at 6 months were either positive at the time of discharge but not sustained or were negative at the time of discharges (runaway, detention, psychiatric hospital or transfer to another residential program). DCFS is Department of Children and Family Services. Non-DCFS is all other funding sources including Department of Human Services, the state school board of education, the state department of corrections, and a neighboring state’s funding for mental health services

While the lack of a family resource seems the obvious source of difference between working with the youth and families with DCFS funding, discharges to “step-downs” in level of treatment (group homes, transitional living programs and more moderate residential care) also resulted in disrupted favorable discharges. This lack of continuity in placement suggests that in some way, the REStArT model’s treatment was differentially effective for youth with different funding sources even though both sets of youth and their outside resources took part in family-oriented consultations at rates that were, practically speaking, very similar.

### Qualitative

Across examples of unsuccessful DCFS cases, therapists noted that youth often wanted to live with a family member but were discouraged from doing so. Youth were either informed by a DCFS caseworker that their chosen family members were not appropriate as a discharge resource or the youth and their family members perceived undue barriers placed on family members that interfered with the youth being placed in their care. Sometimes therapists spoke of this broadly, such as a case in which a therapist noted, “The youth had other family in state but there was little momentum from the system to pursue those avenues.” In other cases, the therapist’s view of the youth’s barrier was more specific: “The expectation was that they [any identified family member] come visit him on campus several times and then she [the DCFS caseworker] would talk about home visits.” In both of these cases the youth ran away from treatment to be with family. Another therapist discussed a case in which a youth was detained by corrections which resulted in his discharge. “After dealing with his legal issues, he went to live with family.”

Therapists commented on cases in which the initial discharge was favorable, a group home or transitional living program, but ultimately the placement was not sustained. One therapist observed: “Sometimes DCFS youth remain [in treatment] for years, and so when a group home is finally suggested…the client is just desperate to leave, and agrees to the goal…in reality they were hoping all along they could go to a parent.” In a specific example of this, one therapist reflected on a case in which a client wanted to return to family in a different part of the state. When a group home was offered, the youth agreed with the expectation that the group home would be closer to his family. It was not, and his behaviors deteriorated once he was placed in the group home. The first principle of the REStArT model is the development of a working therapeutic alliance with the family and youth that includes youth- and family-driven goals. These examples suggest that, at times, it is possible that the model was not being implemented as intended in that the goals identified by the youth and family were not accepted by treatment providers or collaterals.

Therapists also reflected on factors internal to the agency that may impact the success of DCFS-funded cases. The theme that emerged was one in which fidelity to the model’s emphasis on the recognition of ambivalence during consultation may be abdicated when DCFS is involved. Therapists and other treatment providers may see the responsibility of finding an appropriate discharge as the DCFS caseworker’s responsibility. One therapist observed that “we come up against a natural split with DCFS” in which DCFS staff may advocate one discharge plan and treatment providers may advocate another. In the process, the youth’s treatment needs tend to get overlooked because families may simply choose to align with whomever will support their discharge goal. If this split is left unacknowledged in the consultation process, the discharge is less likely to be stable. In another case, a youth was discharged to a former foster home, but then soon re-hospitalized. The therapist thought that “maybe we were pushing too hard and not hearing her [the foster mother] or her concerns and that is why there was not as good as a partnership as there could have been in the treatment process.” Therapists seem to be identifying ways in which the principles of “finding imbalances in the system” and “working with ambivalence” were neglected in these cases.

Not surprisingly, for DCFS cases that were successful, therapists noted that agency providers were able to work cooperatively with DCFS caseworkers, the youth’s biological family, and the youth. In particular, they observed that the youth were given the opportunity “to drive that conversation.” In one case described by a therapist, a youth was encouraged to find a family member who “was willing to participate in treatment and the youth was discharged to them.” This resonates with other studies that have emphasized more than simple contact between youth and families, but rather the development of collaboration and partnerships between providers and families in order to work toward their goals (Geurts et al. 2012; Scarborough et al. 2013; Sharrock et al. 2013).

The lack of emphasis on finding family members by an agency such as DCFS that is charged with protecting children from potential unsafe family situations is understandable. It is notable, however, that the average age of discharge for these youth was just under 17. One of the therapists shared the case of a youth who stated that he intended to leave treatment when he was 18 in order to reunite with family, whether or not his move was supported by treatment providers. As a recent literature review suggests (Collins et al. 2008), the developmentally appropriate focus on knowing one’s place with family is a significant aspect of adolescence, so treatment that acknowledges and preserves those relationships seems likely to be successful in a more enduring way. The successful cases therapists noted appeared to preserve these family relationships, which is consistent with the model’s principle that asks providers to “expect health” from youth and their families.

In investigating outcomes related to family involvement and the REStArT model, we were interested in answering the question, “Do youth and their families benefit from treatment when they leave and is that benefit enduring?” Most of the youth who left the agency’s residential program were older adolescents living in a less restrictive placement. The durability of the treatment’s impact, however, varied depending on the youth’s guardianship. Those youth whose families were involved with DCFS were much more likely to leave without completing treatment or to have favorable discharges that disrupted. When therapists’ reflected on the process of individual cases, both positive and negative, they suggested that the model’s implementation was challenged in a number of ways, such as the development of a therapeutic alliance in which youth and their families were given ownership over their goals. These results suggest on the one hand that, in response to the need for family-driven treatment to improve residential outcomes, the REStArT model offers one way of successfully responding to families’ needs. But the results also suggest that families with DCFS involvement may not be receiving the same benefits of the model’s efforts at collaboration.

## Limitations and Implications for Research and Treatment

A number of limitations need to be acknowledged that temper the interpretation of these results. While it appears that the model’s emphasis on family involvement has differential effectiveness based on the family’s relationship with DCFS, this current study examined only 1 year of discharges so it is possible that this sample of youth with DCFS guardianship is different than other years. Indeed, previous research (McConnell and Taglione 2012) found more comparable results at least at the time of discharge even in returns to family homes between youth with different funding sources. However, the previous study did not consider the sustainability of favorable outcomes.

One possible hypothesis for why relatively equal frequency of family interventions was less successful with DCFS-funded youth relates to the implementation of the model. The therapists interviewed speculated that the fidelity of the model was compromised in cases that included DCFS involvement. Another limitation then of this current study is that it did not contain a fidelity check that would allow us to see if, in fact, therapists and other treatment providers were not implementing the model as it is intended. Without such a check, it is unclear if the model was implemented accurately but was less effective in these cases or if the model was not being used as intended.

This study looked only at whether discharges that were favorable at the time of discharge were sustained. A number of therapists observed anecdotally that DCFS-funded youth sometimes ran away from treatment and returned to live with family in response to being limited in their family contact by treatment providers, DCFS or the courts. An additional limitation then of this study is that there may be youth who were able to return successfully to family homes despite external prohibitions.

These limitations also suggest important steps both in terms of research and, based on greater understanding, policy and treatment, as well. It would be useful to continue to track the trajectories of youth with DCFS funding in order to understand if their outcomes are less often sustained than youth who have a family guardian. Additionally, a careful process review of what happens in treatment of positive and negative outcomes would help to illuminate more specifically the aspects of family involvement that are most likely to help youth and their families and what gets in the way of this process so that barriers to effective treatment can be removed. If, in fact, youth are sometimes running away to live with family, it would be helpful to look at whether these self-directed discharges are successful and if so, what prevented treatment providers from working with the youth and the family proactively.

The need for family involvement in residential treatment has been soundly supported by research and increasingly accepted by residential treatment programs. However, the effectiveness of family interventions will remain limited if providers and the larger system working with youth remain ambivalent about youth returning to their families. There is some research to support therapists’ assumption that youth sometimes return to their families when they are old enough to make their own choice (Collins et al. 2008). The REStArT model asks treatment providers to help youth and families address their ambivalence about their relationships with one another but likely needs to do more to address the ambivalence in the larger system.

## Appendix

### Principles: The Relational Re-enactment Systems Approach to Treatment (REStArT)

- *I. Developing a Working Therapeutic Alliance*: Client, family, and service providers agree on the goals and tasks of treatment. These goals and tasks need to be youth and family driven.
- *II. Relational Re*-*Enactment*: Identify youth’s attachment style through the ways in which the youth re-enacts it in his/her behavior with others (i.e., identify the conflict cycle).
- *III. Managing Counter*-*Response*: Identify the adult counter-response (feelings and subsequent behavior) within that youth’s particular conflict cycle; identify the adult’s unpleasant reality (related to the youth’s conflict cycle) that is being avoided by the adult; face the adult’s unpleasant reality and the adult’s feelings so that they are not driving the adult’s behavior (counter-response).
- *IV. Systems*-*Oriented*: Identify all the adults involved with the youth and have them come together to develop a shared understanding of and way of approaching the youth.
- *V. Finding the Imbalance in the System*: Identify polarities in youth’s behavior and subsequent polarities in adults’ counter-response (i.e., splits/divisions within the system).
- *VI. Seeing the Whole Youth*: Identify ways in which our view of the youth has been compartmentalized (i.e., sees the youth in a particular way). Work together and dialogue so that all parties see both sides of the youth—the adaptive side and the maladaptive side.
- *VII. Restoring the Balance*: Use dialogue and consensus to restore balance in developing a plan to interrupt the youth’s conflict cycle (integrate both extremes of the adults’ counter-response reactions in order to arrive at a more balanced response).
- *VIII. Interrupting the Conflict Cycle*: Implement a plan that interrupts the way the youth typically responds to stressors which provides an opportunity for the youth to respond in a new more adaptive way.
- *IX. Working with Ambivalence*: Be aware of and identify examples of ambivalence toward the current circumstance in the family and the youth so that this can be verbalized instead of expressed through behavior.
- *X. Expecting Health*: Trust the youth’s ability to determine their own goals, tolerate disappointments, and repair relational disruptions.
- *XI. Ownership at Every Part of the System*: Create investment in the model across the entire system and support each part’s contribution to the plan, which promotes responsibility and accountability.
- *XII. Evidence*-*Based*: Use concrete data about the youth to determine conflict cycle and plan development and to evaluate effectiveness and outcomes.
- *XIII. Dynamic and Reflexive Process:* Establish a continuous process of looking at our own responses/reactions and evaluating whether the plan is effective.
